# Supplementary material for: Modeling glioblastoma heterogeneity as a dynamic network of cell states
Source: Mol Syst Biol. 2021 Sep 16;17(9):e10105. doi: 10.15252/msb.202010105 (PMC8444284; doi:10.15252/msb.202010105)
Supplement: Supplementary file 6 — Source Data for Figure 5 [file MSB-17-e10105-s004.zip › Figure5A_sourcedata/GSEA_3071/nosigdb_stateVI.GseaPreranked.1622795169056/FELDMAN_2020_LATEG1.html]

Details for gene set FELDMAN\_2020\_LATEG1[GSEA]

|  || Dataset | state63071 |
| Phenotype | NoPhenotypeAvailable |
| Upregulated in class | na\_pos |
| GeneSet | FELDMAN\_2020\_LATEG1 |
| Enrichment Score (ES) | 0.33762732 |
| Normalized Enrichment Score (NES) | 2.198019 |
| Nominal p-value | 0.0 |
| FDR q-value | 0.002392036 |
| FWER p-Value | 0.012 |
Table: GSEA Results Summary

  

Fig 1: Enrichment plot: FELDMAN\_2020\_LATEG1      
 Profile of the Running ES Score & Positions of GeneSet Members on the Rank Ordered List

  

| PROBE | GENE SYMBOL | GENE\_TITLE | RANK IN GENE LIST | RANK METRIC SCORE | RUNNING ES | CORE ENRICHMENT || 1 | SERPINE1 |  |  | 0 | 1.673 | 0.0396 | Yes |
| 2 | CITED2 |  |  | 5 | 1.232 | 0.0677 | Yes |
| 3 | CDC20 |  |  | 15 | 1.073 | 0.0906 | Yes |
| 4 | LAYN |  |  | 25 | 0.991 | 0.1116 | Yes |
| 5 | PRKAG2 |  |  | 40 | 0.913 | 0.1293 | Yes |
| 6 | EDN1 |  |  | 50 | 0.866 | 0.1473 | Yes |
| 7 | ARHGAP29 |  |  | 54 | 0.863 | 0.1670 | Yes |
| 8 | NEXN |  |  | 62 | 0.843 | 0.1850 | Yes |
| 9 | MYC |  |  | 69 | 0.815 | 0.2026 | Yes |
| 10 | GADD45B |  |  | 90 | 0.758 | 0.2150 | Yes |
| 11 | TAGLN |  |  | 100 | 0.737 | 0.2300 | Yes |
| 12 | PRSS23 |  |  | 108 | 0.732 | 0.2454 | Yes |
| 13 | UGCG |  |  | 124 | 0.700 | 0.2578 | Yes |
| 14 | PHLDA2 |  |  | 126 | 0.697 | 0.2740 | Yes |
| 15 | ANKRD1 |  |  | 137 | 0.684 | 0.2874 | Yes |
| 16 | EZR |  |  | 148 | 0.667 | 0.3004 | Yes |
| 17 | PAWR |  |  | 158 | 0.654 | 0.3134 | Yes |
| 18 | MYL12A |  |  | 173 | 0.627 | 0.3244 | Yes |
| 19 | CCNB1 |  |  | 181 | 0.620 | 0.3371 | Yes |
| 20 | PTTG1 |  |  | 344 | 0.514 | 0.3040 | Yes |
| 21 | PLK2 |  |  | 363 | 0.505 | 0.3109 | Yes |
| 22 | CYR61 |  |  | 377 | 0.498 | 0.3191 | Yes |
| 23 | FHL2 |  |  | 397 | 0.491 | 0.3254 | Yes |
| 24 | CTGF |  |  | 404 | 0.487 | 0.3353 | Yes |
| 25 | SORBS2 |  |  | 437 | 0.476 | 0.3376 | Yes |
| 26 | FBXO32 |  |  | 556 | 0.438 | 0.3150 | No |
| 27 | TPM1 |  |  | 574 | 0.432 | 0.3205 | No |
| 28 | ARL4C |  |  | 666 | 0.408 | 0.3048 | No |
| 29 | LBH |  |  | 702 | 0.396 | 0.3043 | No |
| 30 | HERPUD1 |  |  | 830 | 0.371 | 0.2777 | No |
| 31 | KLF6 |  |  | 854 | 0.368 | 0.2799 | No |
| 32 | CCNB2 |  |  | 967 | 0.350 | 0.2569 | No |
| 33 | UBE2S |  |  | 974 | 0.349 | 0.2636 | No |
| 34 | MEST |  |  | 1294 | 0.304 | 0.1816 | No |
| 35 | RAB13 |  |  | 1416 | 0.290 | 0.1547 | No |
| 36 | FDFT1 |  |  | 1552 | 0.274 | 0.1234 | No |
| 37 | KRT18 |  |  | 1570 | 0.272 | 0.1251 | No |
| 38 | BIRC5 |  |  | 1667 | 0.263 | 0.1045 | No |
| 39 | MKI67 |  |  | 1825 | -0.253 | 0.0666 | No |
| 40 | ID3 |  |  | 1835 | -0.254 | 0.0701 | No |
| 41 | SAT1 |  |  | 1952 | -0.271 | 0.0441 | No |
| 42 | CKAP2 |  |  | 2109 | -0.299 | 0.0076 | No |
| 43 | ARID5B |  |  | 2156 | -0.306 | 0.0020 | No |
| 44 | TSC22D1 |  |  | 2193 | -0.313 | -0.0006 | No |
| 45 | STK17A |  |  | 2336 | -0.340 | -0.0322 | No |
| 46 | PBK |  |  | 2412 | -0.356 | -0.0448 | No |
| 47 | SMC4 |  |  | 2634 | -0.408 | -0.0969 | No |
| 48 | CENPF |  |  | 2842 | -0.472 | -0.1435 | No |
| 49 | SOX4 |  |  | 3066 | -0.571 | -0.1923 | No |
| 50 | NUSAP1 |  |  | 3199 | -0.663 | -0.2135 | No |
| 51 | SOX2 |  |  | 3314 | -0.778 | -0.2270 | No |
| 52 | ARL6IP1 |  |  | 3339 | -0.804 | -0.2146 | No |
| 53 | TMEFF2 |  |  | 3344 | -0.808 | -0.1966 | No |
| 54 | TGIF1 |  |  | 3380 | -0.860 | -0.1860 | No |
| 55 | CDK1 |  |  | 3396 | -0.883 | -0.1692 | No |
| 56 | HMGB2 |  |  | 3414 | -0.922 | -0.1522 | No |
| 57 | TOP2A |  |  | 3457 | -1.020 | -0.1397 | No |
| 58 | LIMA1 |  |  | 3564 | -1.432 | -0.1354 | No |
| 59 | BEX1 |  |  | 3608 | -1.883 | -0.1028 | No |
| 60 | SCRG1 |  |  | 3613 | -2.165 | -0.0526 | No |
| 61 | S100B |  |  | 3624 | -2.502 | 0.0039 | No |
Table: GSEA details [plain text format]

  

Fig 2: FELDMAN\_2020\_LATEG1: Random ES distribution      
 Gene set null distribution of ES for **FELDMAN\_2020\_LATEG1**
